# Proteinuria and baseline renal function predict mortality and renal outcomes after sirolimus therapy in liver transplantation recipients

**DOI:** 10.1186/s12876-017-0611-z

**Published:** 2017-04-20

**Authors:** Lung-Chih Li, Chien-Ning Hsu, Chih-Che Lin, Yu-Fan Cheng, Tsung-Hui Hu, Ding-Wei Chen, Chih-Hsiung Lee, Toshiaki Nakano, Chao-Long Chen

**Affiliations:** 1 Division of Nephrology, Department of Internal Medicine, Kaohsiung, Taiwan; 2 Department of Pharmacy, Kaohsiung Chang Gung Memorial Hospital, 123, Ta Pei Road, Niao Sung District, 833 Kaohsiung, Taiwan; 3 Liver Transplant Center, Department of Surgery, Kaohsiung, Taiwan; 4 Department of Diagnostic Radiology, Kaohsiung, Taiwan; 5 Division of Gastroenterology, Department of Internal Medicine, Kaohsiung, Taiwan; 6 Center for Translational Research in Biomedical Sciences, Kaohsiung Chang Gung Memorial Hospital and Chang Gung University, College of Medicine, Kaohsiung, Taiwan; 7 School of Pharmacy, Kaohsiung Medical University, Kaohsiung, Taiwan

**Keywords:** Liver transplant, Sirolimus, Acute kidney injury, Chronic kidney disease, Proteinuria, Renal insufficiency, Immunosuppression

## Abstract

**Background:**

Chronic kidney disease is a significant complication after liver transplantation (LT), but the role of pre-existing renal insufficiency and proteinuria remains unclear among LT recipients receiving sirolimus.

**Methods:**

We assessed the effects of proteinuria and baseline renal function on long-term renal and survival outcomes among 576 LT recipients who received SRL in a medical center between 2005 and 2014. Renal outcomes were the incidences of >50% reduction in their baseline estimated glomerular filtration rate and end stage kidney disease requiring renal replacement therapy. Proteinuria was identified using morning dipstick results (≥30 mg/dL) at baseline and within the first year after the initiation of SRL therapy. A Kaplan-Meier analysis was performed to estimate time to event. Factors associated with the outcomes were determined using the Cox proportional hazards model with a significance level set at *P* <0.05.

**Results:**

During the study period, renal function deteriorated in 135 (25.3%) patients and 68 (11.8%) patients died. Persistent and new onset proteinuria contributed to a high rate of mortality and the deterioration of renal function (both log-rank tests, *P* <0.0001). After adjustments, new onset proteinuria within the first year after the initiation of SRL therapy increased the risk of deteriorating renal function, regardless of baseline estimated glomerular filtration rate. Moreover, pre-existing (hazard ratio = 1.91; *P* <0.001) and new onset diabetes (hazard ratio = 2.34; *P* <0.0001) were significantly associated with new onset proteinuria among SRL users.

**Conclusions:**

These findings support the effective monitoring and early management of the predictable risks for proteinuria among new SRL users in order to delay the progression of renal disease.

## Background

Chronic kidney disease (CKD) is a common complication among liver transplantation (LT) recipients; up to 20% of patients receiving LT progress to CKD stage 4 or 5 at five years post-transplant [1]. The occurrence of CKD after LT has a major impact on graft and patient survival [2].

Despite the various pre- and post-operative factors contributing to the increased CKD in LT recipients, long-term use of calcineurin inhibitors (CNIs) accounts for a major cause of nephrotoxicity [3–5]. Hence, minimizing the nephrotoxicity of immunosuppressive regimens may help decrease the number of patients who develop CKD and the subsequent morbidity and mortality.

Sirolimus (SRL), a potent immunosuppressant which binds to the FK-binding protein and inhibits the activity of the mammalian target of rapamycin (mTOR) [4], has been used as an alternative or adjuvant to CNI-based immunosuppressants in transplant recipients and may offer potential advantages over a CNI-based regimen. First, CNI-related chronic renal impairment may improve after switching to SRL in both renal and liver transplantation [6, 7]. Second, other side effects caused by CNIs, such as neurotoxicity, hypertension, and post-transplant diabetes mellitus, may be avoided with a SRL-based regimen [8]. Furthermore, the anti-proliferative effects of SRL attenuate fibrosis and improve survival in an animal model of cirrhosis [9], which may be associated with a lower incidence of hepatocellular carcinoma (HCC) recurrence [10] and *de novo* neoplasia [11], particularly in LT recipients. Hence, the use of SRL, or other mTOR inhibitors, in LT patients has been increasing in routine clinical practice.

However, evidence shows that not all LT recipients who converted to a SRL-based regimen experienced the benefit of renal function protection. Although many studies have demonstrated that an SRL-based or SRL mono therapy were effective and safe for renal protection in LT patients [7, 12–14], some reported no change [15, 16] or even a worsening [17] of renal function after converting to SRL. To date, the renoprotective mechanism of SRL and reasons for why the switch only benefits some patients, but others, remain inconclusive. In addition, many studies found that proteinuria developed after SRL initiation in liver, as well as, in other organ transplant recipients [14, 18, 19]. Proteinuria is a sensitive marker for CKD progression; however, its implication in LT recipients before and after SRL remains elusive. We examined 576 adult LT recipients who were converted to an SRL-based regimen during 2005-2014 in a medical center. The aim of this study was to investigate the effect of baseline renal function and proteinuria on renal and survival outcomes among LT recipients who were new to treatment with SRL.

## Methods

### Data source and study cohort

This retrospective cohort study was comprised of patients who received a SRL-based immunosuppressive regimen after LT from January 2005 to December 2014 at Kaohsiung Chang Gung Memorial Hospital in Taiwan. Patients were identified using electronic healthcare data that included their medical, medication administration, and procedure records and laboratory results maintained in the study setting. Indications for LT and SRL were found and recorded by reviewing the medical records.

### Outcomes and covariates

The primary study endpoint was the incidence of a >50% reduction in the baseline estimated glomerular filtration rate (eGFR) or the initiation of chronic dialysis (continuously for ≥ 3 months) following the initiation of SRL therapy. The >50% reduction in eGFR was considered an “acute kidney injury” event with a causal association with mortality and progression to chronic renal failure [20]. Secondary endpoints were all-cause mortality and the occurrence of proteinuria within 12 months after the initiation of SRL. The glomerular filtration rate (mL/min/1.73 m^2^), which was used to determine renal function, was estimated using the Modification of Diet in Renal Disease (MDRD) Study formula [21]. Proteinuria was identified using results from a morning dipstick taken at baseline and within 12 months after SRL initiation. Dipsticks that resulted with 1+ protein or above, which is equivalent to ≥ 30 mg/L of proteinuria, were considered positive.

Patients received follow-up from the date of SRL initiation (index date) until either death, December 31, 2014 (last date in the database), or the first event of interest occurred, whichever came first. For the purpose of this study, the following baseline characteristics of each LT recipient were retrieved 12 months prior to or at the index date: demographics (gender, age at the index date), Quan–Charlson Comorbidity Index score [22], transplant-related variables and laboratory test results, immunosuppressive regimens before and after SRL initiation, serum trough levels (ng/mL) at < 3, 3-6, and 6-12 months after SRL initiation.

### Statistical analysis

Categorical data were summarized as counts and proportions. Means with standard deviation (SD) were used to describe continuous data. Kaplan-Meier analysis and a log-rank test were performed to estimate time to the first evaluated event after the initiation of SRL. The effect of proteinuria on the deterioration of renal function were estimated using the Cox proportional-hazards model, controlling for patient’s characteristics and clinical conditions, to determine the hazard ratio (HR) with a 95% confidence interval (CI). A stratified analysis was performed in order to identify the relative risk of proteinuria on renal outcomes between patients with eGFR ≥ 60 and < 60 mL/min/1.73 m^2^ at baseline. Differences with a *P* < 0.05 were considered significant. Processing and analyses of data were conducted using SAS Enterprise Guide v 5.1 (SAS, Cary, NC, USA).

## Results

### Patient characteristics

In total, 576 adult LT recipients received SRL between January 1, 2005 and December 31, 2014. At the time of SRL initiation, the majority of patients (*n* = 520) were receiving tacrolimus (*n* = 508) or cyclosporine (*n* = 12). The characteristics of the study cohort are shown in Table 1. The mean age at SRL initiation was 54 years (53.93 ± 9.2), 79% (*n* = 456) of the LT patients were male adults, and the mean duration between LT and SRL initiation was 15.67 months (±33.5). The mean baseline eGFR was 78.6 (±42.94) ml/min/1.73 m^2^ and 14.02% of them had proteinuria at baseline. The major indications for LT included, but were not mutually exclusive to, liver cirrhosis related to HCC (55.21%), hepatitis B virus (HBV) (55.03%), and hepatitis C virus (HCV) (28.13%). One-fourth of the study cohort had a history of diabetes mellitus and 3.8% of LT recipients had CKD or required chronic dialysis prior to SRL therapy.

**Table 1:** Characteristics of the study cohort (*n* = 576)

Characteristics	Data
Age at baseline, years	53.93 ± 9.20
Sex, male, %	456 (79.17%)
Duration between LT and SRL initiation, months	15.70 ± 33.5
eGFR at baseline, ml/min/1.73 m^2^	78.60 ± 42.94
SCr at baseline, mg/dL	1.16 ± 0.72
Positive proteinuria at baseline (*n* = 535), n (%)	75 (14.02%)
Indications for liver transplantation, n (%) ^a^	
Decompensated liver cirrhosis	
Hepatitis B	317 (55.03%)
Hepatitis C	162 (28.13%)
Alcoholic	87 (15.10%)
HCC	318 (55.21%)
Acute liver failure	34 (5.9%)
Baseline comorbidities, n (%)	
Congestive heart failure	3 (0.52%)
Diabetes mellitus	144 (25%)
Celebrovascular disease	5 (0.87%)
Chronic pulmonary disease	26 (4.52%)
Renal disease	21 (3.65%)
CNI use at baseline	520 (90.28%)
Plasma trough level of SRL, ng/mL	
<3 months,	6.19 ± 3.52
3-6 months	6.26 ± 2.96
6-12 months	6.19 ± 2.88
Duration of follow-up, day, (*n* = 574)	1075 ± 871.2

Data presents as mean ± SD for continuous data, and n (%) for categorical data

^a^Not excluding events

The mean follow-up duration was 2.9 years (1075 ± 871.2 days). During the study period, 3 (0.19%) patients were newly diagnosed with ESRD, they received dialysis therapy and 85 (10.5%) patients developed new onset proteinuria during the first year of follow-up. There were 68 patient deaths (mortality rate = 11.8%) and HCC (*n* = 20) was the leading cause of death (not listed in the table).

By reviewing the medical records, we compiled the indications for SRL therapy in our study cohort, which are listed in Table 2. The major reasons for SRL initiation included renal dysfunction (55%), prophylaxis for HCC recurrence (16.1%), and acute/chronic rejection (14.6%).

**Table 2:** Indications of sirolimus therapy

Variable	N (%)
Renal dysfunction^a^	318 (55.2)
HCC	
Prophylaxis	93 (16.1)
Recurrence	21 (3.6)
Non-hepatic tumors	18 (3.1)
Biopsy proved acute/chronic rejection	84 (14.6)
Neurotoxicity	5 (0.9)
Other side effects from immunosuppressants	37 (6.4)

^a^Defined by physician’s criteria or eGFR <60 mL/min/1.73 m^2^ without other reasons for SRL use

### Effects of baseline proteinuria on the deterioration of renal function and mortality

**Fig. 1:**
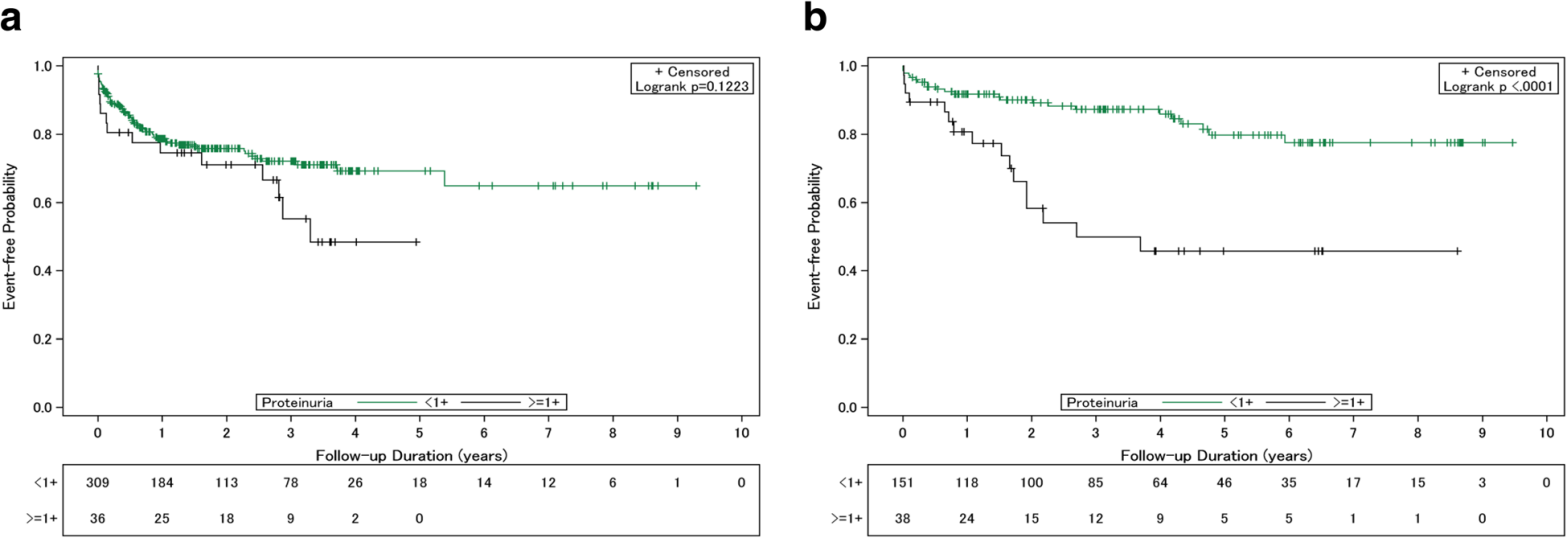
Patient renal outcomes based on the presence of baseline proteinuria. **a** Liver transplant (LT) recipients with a baseline estimated glomerular filtration rate (eGFR) ≥60 ml/min/1.73 m^2^, **b** LT recipients with baseline eGFR <60 ml/min/1.73 m^2^. <1+, without, or undetectable proteinuria (<30 mg/dL); ≥1+, with positive result of proteinuria

A Kaplan-Meier analysis with the log-rank test was performed in order to assess the influence of baseline proteinuria on the time to progression of renal disease after LT (Fig. 1). Among the patients who had an eGFR ≥60 ml/min/1.73 m^2^ (*n* = 345), the cumulative risk for worsening renal outcome was not significant between patients with and without proteinuria at baseline (*P* = 0.12) (Fig. 1a). In contrast, among patients with eGFR <60 ml/min/1.73 m^2^ (*n* = 189), the incidence of renal disease progression was significantly higher in SRL users with baseline proteinuria when compared to those with negative or undetectable proteinuria (*P* <0.0001) (Fig. 1b). The cumulative risk for renal disease progression was higher in patients with baseline proteinuria than in those without in the first (18.42% vs 7.95%, respectively), third (39.47% vs 11.26%, respectively), and fifth (42.11% vs 14.57%, respectively) years of follow-up.

**Fig. 2:**
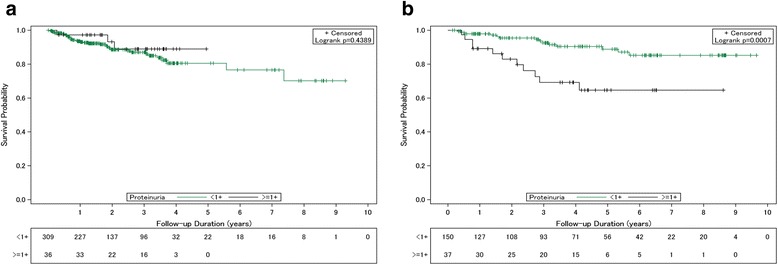
Patient survival outcome based on the presence of baseline proteinuria. **a** Liver transplant (LT) recipients with a baseline estimated glomerular filtration rate (eGFR) ≥ 60 ml/min/1.73 m^2^, **b** LT recipients with baseline eGFR < 60 ml/min/1.73 m^2^. <1+, without, or undetectable proteinuria (<30 mg/dL); ≥1+, with positive result of proteinuria

The association between baseline proteinuria and all-cause mortality is presented in Fig. 2. In patients with an eGFR ≥60 mL/min/1.73 m^2^, there was no significant difference of mortality between patients with and without baseline proteinuria (*P* = 0.44) (Fig. 2a). However, in patients with an eGFR <60 mL/min/1.73 m^2^, patients with proteinuria at baseline had a significantly lower survival rate (*P* <0.001) than patients without proteinuria at baseline (Fig. 2b). The cumulative risk for mortality was higher in patients with baseline proteinuria than in those without proteinuria in the first (10.81% vs 2.0%, respectively), third (27.03% vs 6.0%, respectively) and fifth (29.73% vs 8.0%, respectively) years of follow-up.

### Effects of new onset proteinuria on deterioration of renal function and mortality

**Fig. 3:**
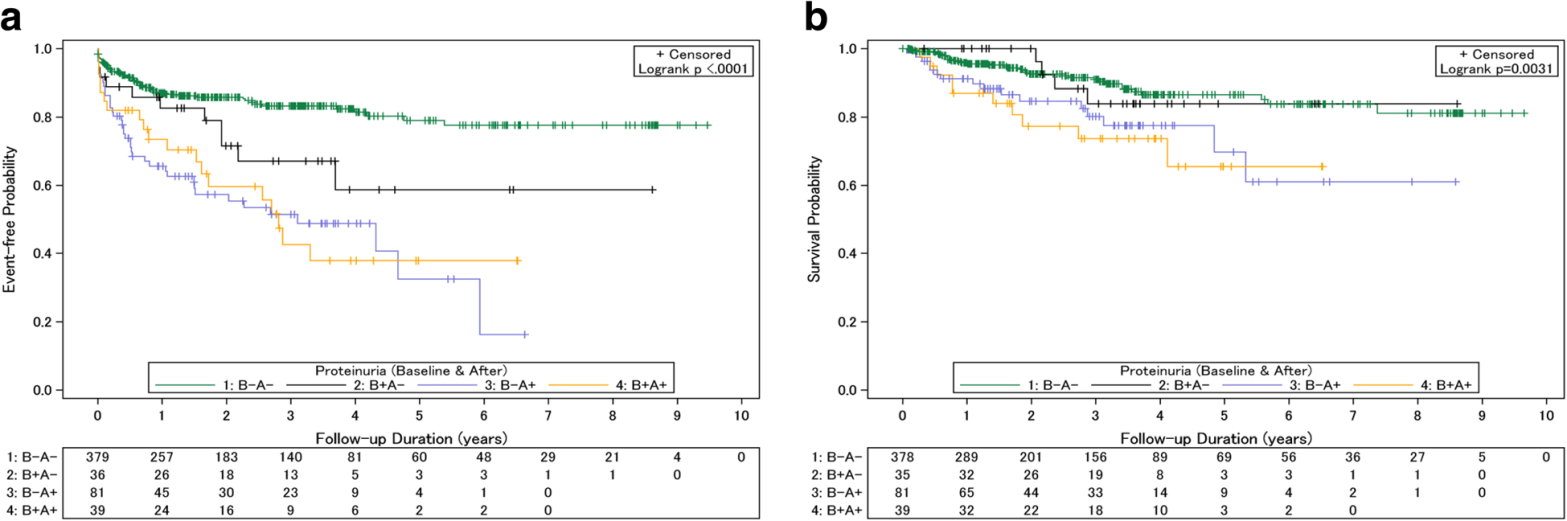
Patient outcome based on the presence of proteinuria before and after sirolimus (SRL) initiation. **a** Renal outcome, **b** Survival outcome. B-A-, patients without proteinuria before (B-) and after (A-) SRL initiation; B + A-, patients with proteinuria before (B+) but not after (A-) SRL initiation; B-A+, patients without proteinuria (B-) but with new onset proteinuria after (A+) SRL initiation; and B + A+, patients with persistent proteinuria before (B+) and after (A+) SRL initiation

In the study cohort, 85 (18.6%) patients developed new onset proteinuria within the first year of SRL therapy. In order to determine when proteinuria occurred and its impact on renal outcome, the patients were categorized into the 4 following groups based on the presence (+) or absence (-) of proteinuria before (B) and after (A) SRL initiation for the comparison analysis. Patients categorized as B + A+ (*n* = 39) and B-A+ (*n* = 81) were found to be associated with the poorest renal outcome, followed by patients with B + A- and B-A- (Fig. 3a). The cumulative rate of renal dysfunction at 5 years was 48.72%, 46.91%, 30.56%, and 15.30% in patients categorized as B+A+, B-A+, B+A-, and B-A-, respectively. As shown in Fig. 3b, mortality was high among patients with persistent proteinuria (B + A+) and new-onset proteinuria (B-A+). The 5-year cumulative mortality rate was 25.64%, 18.52%, 11.43%, and 8.2% in patients categorized asB+A+, B-A+, B+A-, and B-A-, respectively.

To explore the potential factors of an increased risk for new onset proteinuria after SRL exposure, a multivariate regression analysis was conducted to determine the hazard ratios (HR) for proteinuria onset during the post-SRL period (Table 3). After adjustment, the development of proteinuria during follow-up was significantly associated with pre-existing diabetes mellitus (HR = 1.91; 95% CI = 1.32-2.77; *P* <0.001) and new onset diabetes mellitus developed during the follow-up (HR = 2.34; 95% CI = 1.62-3.38; *P* < 0.0001).

**Table 3:** Factors associated with new onset proteinuria^a^ (*n* = 455)

	Crude		Adjusted	
Variable	HR (95%CI)	*P value*	HR (95%CI)	*P value*
Age at initiation, year				
50-64 vs 17-49	1.16 (0.76 − 1.75)	0.04	1.05 (0.68 − 1.62)	0.84
≥65 vs 17-49	2.22 (1.19 − 4.15)	0.04	1.75 (0.90 − 3.39)	0.10
Male vs female patients	0.61 (0.42 − 0.90)	0.02	0.67 (0.42 − 1.06)	0.09
Baseline eGFR < 60 vs ≥60 mL/min/1.73 m^2^	1.62 (1.15 − 2.27)	0.006	1.28 (0.89 − 1.85)	0.18
Hepatitis B	1.02 (0.72 − 1.44)	0.63	1.23 (0.78 − 1.95)	0.37
Hepatitis C	1.30 (0.90 − 1.87)	0.02	1.26 (0.80 − 1.97)	0.32
Alcoholism	0.64 (0.36 − 1.14)	0.74	0.79 (0.42 − 1.47)	0.45
Decompensated liver cirrhosis	1.24 (0.89 − 1.74)	0.20	1.21 (0.84 − 1.74)	0.32
HCC	1.02 (0.73 − 1.44)	0.29	1.06 (0.72 − 1.57)	0.76
Diabetes mellitus	2.32 (1.64 − 3.28)	<.0001	1.91 (1.32 − 2.77)	<.001
New onset diabetes mellitus^b^	2.77 (1.96 − 3.90)	<.0001	2.34 (1.62 − 3.38)	<.0001
CNI prior use	2.30 (0.73 − 7.27)	0.15	2.27 (0.69 − 7.43)	0.18
CNI use after	0.69 (0.32 − 1.48)	0.33	0.67 (0.29 − 1.54)	0.35
SRL trough level < 3 months	0.97 (0.91 − 1.03)	0.30	1.01 (0.95 − 1.07)	0.81

^a^Event of new onset proteinuria was identified within 1 year during the follow-up among a subgroup of patients without proteinuria presence at baseline

^b^New onset diabetes mellitus anytime in the follow-up period

### Additional risk factors associated with deterioration of renal function

Table 4 shows factors associated with renal outcome by baseline eGFR. The development of new proteinuria after the initiation of SRL was a strong independent risk factor for renal dysfunction among patients with a baseline eGFR <60 ml/min/1.73 m^2^ (HR = 5.38; 95% CI = 2.49-11.62; *P* < 0.0001) and with a baseline eGFR ≥60 ml/min/1.73 m^2^ (HR = 3.12; 95% CI = 1.92-5.09; *P* < 0.0001). Although this difference was not statistically significant, the presence of baseline proteinuria demonstrated to increase a 93% (*P* = 0.11) and 29% (*P* = 0.42) greater risk for worsening renal outcome. Among patients with a baseline eGFR ≥60 ml/min/1.73 m^2^, men (HR = 0.35; 95% CI = 0.19-0.65; *P* = 0.001) were less likely to have progression of renal dysfunction, while those with a history of HCC (HR = 1.85, 95% CI = 1.03-3.29; *P* = 0.04) were more likely.

**Table 4:** Factors associated with deterioration of renal function^a^ (*n* = 533)

Variable	Baseline eGFR < 60 mL/min/1.73 m^2^		Baseline eGFR ≥ 60 mL/min/1.73 m^2^	
	Adjusted HR (95%CI)	*P value*	Adjusted HR (95%CI)	*P value*
Age at initiation, year				
50-64 vs 17-49	0.77 (0.29 − 2.05)	0.60	1.14 (0.66 − 1.95)	0.64
≥65 vs 17-49	1.08 (0.23 − 5.13)	0.92	1.20 (0.45 − 3.16)	0.72
Male vs female patients	0.82 (0.36 − 1.84)	0.61	0.35 (0.19 − 0.65)	0.001
Prior hepatitis B	0.48 (0.21 − 1.09)	0.08	0.83 (0.45 − 1.55)	0.56
Prior hepatitis C	1.43 (0.61 − 3.37)	0.40	0.93 (0.51 − 1.69)	0.80
Prior alcoholism	1.52 (0.61 − 3.77)	0.37	1.35 (0.72 − 2.54)	0.35
Prior decompensated liver cirrhosis	1.04 (0.50 − 2.17)	0.92	1.55 (0.97 − 2.46)	0.07
Prior HCC	0.66 (0.32 − 1.39)	0.28	1.85 (1.03 − 3.29)	0.04
Prior diabetes mellitus	0.95 (0.43 − 2.09)	0.89	1.20 (0.71 − 2.01)	0.50
New onset diabetes mellitus	1.47 (0.69 − 3.16)	0.32	0.63 (0.40 − 1.24)	0.23
Proteinuria at baseline	1.93 (0.87 − 4.29)	0.11	1.29 (0.70 − 2.39)	0.42
New onset proteinuria < 1 year	5.38 (2.49 − 11.62)	<.0001	3.12 (1.92 − 5.09)	<.0001

^a^Event of renal function reduction was either eGFR declined > 50%, start receiving chronic dialysis or renal transplantation; whichever comes first

## Discussion

The study demonstrates that pre-existing renal insufficiency and proteinuria appear to be associated with an increased incidence of renal function deterioration and mortality among LT recipients who received SRL. New onset proteinuria within the first year of SRL therapy was a strong independent risk factor for worsening renal function, regardless of baseline renal function at the time of SRL initiation. Diabetes, including pre-existing and newly diagnosed, was associated with the development proteinuria after SRL initiation in all LT recipients.

mTOR inhibitors have been widely used in LT recipients, not only for reducing the risk of allograft rejection and neoplasm, but also for renal protection. Because pre-LT renal disease is a well-recognized risk for many adverse outcomes, including the need for dialysis and poor patient survival, it is important to investigate the renal protective role of SRL and risk factors associated with the progression of renal disease in LT recipients.

Our study demonstrated that a higher mortality rate and faster renal function decline was associated with baseline proteinuria and impaired renal function (eGFR <60 ml/min/1.73 m^2^). There is little evidence showing the significance of baseline proteinuria at SRL initiation on renal function in LT patients. Most of the experience is with renal transplantation recipients. One study reported that proteinuria that was <800 mg/day was significantly associated with a better renal outcome in kidney transplant recipients who converted to SRL therapy [23]. Results from another study demonstrated a negative correlation between baseline proteinuria and rate of graft rejection and survival after kidney transplantation [24]. Despite the lack of statistical power in the present study, LT recipients with a baseline proteinuria trended towards poor long-term renal function, which is consistent with previous studies [23, 24].

The presence of proteinuria within the first year of SRL therapy revealed an important role in predicting the progression of renal function and death in our study cohort. There are studies indicating that mechanisms for SRL-induced proteinuria include podocyte degeneration, alteration in the glomerular expression of vascular endothelial growth factor, and reduced tubular protein reabsorption [25–28]. However, the significance of proteinuria after SRL use remains poorly understood.

New onset or the worsening of proteinuria may accelerate the rate of renal function progression. The effect of developing proteinuria on renal outcome in SRL users could be influenced by baseline renal function at the time of the initiation of SRL. For instance, data on 148 LT recipients who converted to SRL shows that those with a proteinuria level >100 mg/dL increased from 45% pre-SRL to 72% at 6 months, 63% at 3 years, 58% at 5 years, and 79% post-SRL. However, there was no significant change in eGFR (pre-SRL, 58.9 ± 28.8 mL/min/1.73 m^2^) in patients with new onset proteinuria after a median follow-up of 1,343 days [12]. Another study examined 102 LT recipients with deteriorating kidney function (mean eGFR = 40.8 ± 16.7 mL/min/1.73 m^2^) prior to initiating SRL, with a median 3.1-year follow-up [29]. The results from this study suggests that new onset proteinuria (≥1000 mg/day) after SRL conversion is an independent risk factor for the deterioration of kidney function (odds ratio = 3.3; 95% CI = 1.1-9.5; *P* = 0.03). The difference in baseline eGFR of those who converted to SRL in the two studies may explain the different renal outcomes observed and also echo our findings that there may be a renal threshold beyond which SRL may not provide benefit.

In order to maximize the number of cases, we recruited patients using a morning urine dipstick to evaluate for proteinuria prior to quantifying spot urine protein in our institute since 2012. Of note, urine dipstick is not as sensitive or specific as other modalities such as the urine albumin to creatinine ratio (UACR) or daily protein measurement. Detection bias and under-diagnosis of proteinuria could lead to an underestimate of the rate of positive proteinuria results. A previous study [30] agrees that 1+ proteinuria detected by urine dipstick is approximately equivalent to a UACR of 30-300 mg/g, which is considered microalbuminuria. Based on this, microalbuminuria that occurs before or within one year of the initiation of SRL may still have greatly increased the risk of worsening renal function. These study results suggest that it is necessary to monitor regularly for proteinuria and renal function pre- and post-SRL initiation, as well as re-evaluating the benefits and risks of continuously taking SRL during post-transplant care. Further study is needed for quantifying the threshold of proteinuria that causes irreversible renal function deterioration.

The presence of pre- and post-LT diabetes are recognized risk factors for CKD and mortality in patients undergoing LT [31, 32]. New onset diabetes after transplantation was associated with the use of immunosuppression. New onset diabetes associated with SRL-based combination therapy was reported in a large group (*n* = 20,124) of kidney transplantation recipients [33]. Lamming et al. [34] demonstrated that chronic SRL use can cause insulin resistance through the deactivation of mTOR Complex 2 (mTORC2), which supports the observation of SRL-associated diabetes in kidney transplantation recipients. Wadei et al. reports that post-LT diabetes was associated with a 3.6-fold increase of new onset proteinuria after SRL in a LT setting [29]. Furthermore, both pre-existing and new onset diabetes were found to be the main causes of new onset proteinuria within 1 year after SRL initiation in our study cohort.

Regardless of immunosuppression uses, non-alcohol steatohepatitis (NASH) have been demonstrated to be associated with a higher prevalence (OR, 2.53; 95% CI, 1.6-4.1) and incidence (HR 2.12, 95%CI 1.4-3.2) of CKD than simple steatosis in a systematic review and meta-analysis of 33 studies involving 64,000 individuals [35]. Although the association between NASH and CKD was strong from cross-sectional studies, the causal relationship between NASH and CKD remains debatable [36]. Unlike NASH is increasing prevalent etiology for liver transplantation in Western countries [37], the leading indication for LT has been viral hepatitis in Asia-Pacific region for decades, including Taiwan, Japan and South Korean [38]. Although the impact of NASH on renal outcomes is not apparent in the present study, both baseline type 2 diabetes (25%) and eGFR < 60 ml/min/1.73 m^2^ (35%) are prevalent in our study cohort, which suggests that it is equally important to prevent the deterioration of renal function before and after transplantation.

**Fig. 4:**
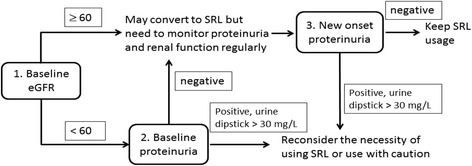
Clinical decision-making algorithm for initiating sirolimus therapy. Before converting to SRL, check the baseline eGFR and proteinuria. If baseline eGFR ≥60 or <60 mL/min/1.73 m^2^ without baseline proteinuria, initiation of SRL can be considered. Among patients with baseline eGFR <60 mL/min/1.73 m^2^ and positive proteinuria, conversion to SRL requires careful evaluation. During SRL therapy, regular monitoring of the eGFR and for proteinuria is required. For patients with new onset proteinuria, re-evaluation of the necessity of SRL, switching immunosuppression regimens, or SRL discontinuation may be considered

Although the renoprotective affect of SRL is supported by findings in basic research, there remains an uncertain gap between these findings and evidence derived from clinical LT practice. This large cohort of adult LT recipients receiving SRL therapy enabled us to demonstrate the role of proteinuria in the prediction of renal outcome, which will help fill the gap. These study results also emphasize the necessity of regularly monitoring for proteinuria and the serum creatinine levels pre- and post-SRL initiation in order to modify the risk for further renal function deterioration early and appropriately. Based on the findings in the current study, a decision-making algorithm was proposed for SRL therapy (Fig. 4). The benefits and risks of initiating SRL must be assessed carefully among patients with renal dysfunction and proteinuria in order to determine its impact on renal outcome. In addition to baseline proteinuria, it is necessary to re-evaluate the appropriateness of SRL continuation among patients who develop proteinuria after the initiation of SRL therapy. Discontinuation of SRL combination therapy and therapeutic interventions, such as renin-angiotensin-aldosterone system blockade, to reduce severity of proteinuria in order to improve renal outcomes should be considered. To prevent deterioration of renal function, more research is needed to determine the duration of treatment and when, and at what level of eGFR, should SRL be initiated.

There were certain limitations to this single-center observational study. Data collection was highly dependent on available information in our routine practice setting. For instance, 24-h urinary protein excretion was not applicable in all patients at regular follow-up. The study results could be biased from missing data and unmeasured confounders. It is difficult to determine whether the new onset proteinuria was caused by SRL use in patients with underlying diabetic nephropathy or newly diagnosed diabetes. The results of this study suggested that the baseline risk of developing new diabetes after SRL therapy warrants further attention in evaluating renal function progression. Having a comparable group of patients who were on non-sirolimus therapy would strength the causal relationship between SRL use and renal outcomes. The generalizability of this study is limited by the etiology of chronic liver disease and baseline characteristics of LT recipients in the study population.

## Conclusions

This study indicated that baseline renal function, proteinuria, and diabetes and post-LT diabetes are important for assessing the protective role of SRL in renal dysfunction. Baseline eGFR and new onset proteinuria within the first year of SRL therapy were independent predictors of renal function progression. Both pre-existing and new onset diabetes were significantly associated with the development of new proteinuria after SRL use. These study findings enable us to develop an effective prevention strategy targeting ESRD risk modification in the clinical practice of LT.

## Abbreviations

CKD: Chronic kidney disease
CNI: Calcineurin inhibitor
eGFR: Estimated glomerular filtration rate
ESRD: End stage of renal disease
HBV: Hepatitis B virus
HCC: Hepatocellular carcinoma
HCV: Hepatitis C virus
HR: Hazard ratio
LT: Liver transplantation
mTOR: Mammalian target of rapamycin
NASH: Non-alcoholic steatohepatitis
SCr: Serum creatinine
SRL: Sirolimus
